# Acclimation of Culturable Bacterial Communities under the Stresses of Different Organic Compounds

**DOI:** 10.3389/fmicb.2018.00225

**Published:** 2018-02-19

**Authors:** Hui Wang, Shuangfei Zhang, Amit Pratush, Xueying Ye, Jinli Xie, Huan Wei, Chongran Sun, Zhong Hu

**Affiliations:** Department of Biology, College of Science, Shantou University, Shantou, China

**Keywords:** pyrene, estrogens, functional bacteria, phylotypes, network

## Abstract

The phylogenetic diversity of bacterial communities in response to environmental disturbances such as organic pollution has been well studied, but little is known about the way in which organic contaminants influence the acclimation of functional bacteria. In the present study, tolerance assays for bacterial communities from the sediment in the Pearl River Estuary were conducted with the isolation of functional bacteria using pyrene and different estrogens as environmental stressors. Molecular ecological networks and phylogenetic trees were constructed using both 16S rRNA gene sequences of cultured bacterial strains and 16S rRNA gene-based pyrosequencing data to illustrate the successions of bacterial communities and their acclimations to the different organic compounds. A total of 111 bacterial strains exhibiting degradation and endurance capabilities in response to the pyrene estrogen-induced stress were successfully isolated and were mainly affiliated with three orders, *Pseudomonadales*, *Vibrionales*, and *Rhodobacterales*. Molecular ecological networks and phylogenetic trees showed various adaptive abilities of bacteria to the different organic compounds. For instance, some bacterial OTUs could be found only in particular organic compound-treated groups while some other OTUs could tolerate stresses from different organic compounds. Furthermore, the results indicated that some new phylotypes were emerged under stresses of different organic pollutions and these new phylotypes could adapt to the contaminated environments and contribute significantly to the microbial community shifts. Overall, this study demonstrated a crucial role of the community succession and the acclimation of functional bacteria in the adaptive responses to various environmental disturbances.

## Introduction

Bacteria can adapt to a range of different habitats, including contaminated and extreme environments, and they can perform a wide diversity of physiological activities (Dash et al., 2013). In terms of pollution, bacteria can play both positive and negative roles in an ecosystem, first by degrading organic waste and then by spreading the resultant dangerous contaminants. They have been well acknowledged as central players in the health of all organisms and ecosystems (Layeghifard et al., 2016). Considering their extensive capabilities for degrading organic contaminants, bacteria have been widely considered for bioremediation in different polluted environments. For instance, Mishamandani et al. (2016) investigated the bacterial community responses associated with the cosmopolitan marine diatom (*Skeletonema costatum*) used to counteract crude oil pollution. The results demonstrated that hydrocarbonoclastic bacteria in the phycosphere of phytoplankton could contribute significantly to biodegradation of hydrocarbon contaminants in marine surface water. McDevitt-Irwin et al. (2017) explained the roles of microbial communities present in coral reefs and the way in which they behave or respond in the presence of various stresses (such as climate change, pollution, and overfishing) generated by the external environment. It was observed that these stresses could lead to a rise in disease-associated bacterial populations in the coral environment. In another example of adaptation, microbial strains under high-stress environments [polycyclic aromatic hydrocarbon (PAH) and steroid contamination] began growing, and were able to use these stress-producing substances as energy and carbon sources (Haritash and Kaushik, 2009).

In the natural environment, microbial communities are not only a simple collection of independent individuals but also exhibit a complex interconnection between different microbial taxa found in the vicinity. These complex inter-taxa interactions make the collective microbiome more significant and effective than the function of any individual constituent species (Rogers et al., 2013; da Silva et al., 2014). It is observed that ecological interactions within different microbial populations can easily influence microbiome composition as well as host health (Hansen et al., 2015). These ecologically interactive microbial communities might compete for resources or exchange genetic material with hosts (Stecher et al., 2012). In addition, some of the inter-taxa interactions or relationships may be beneficial for adapting different populations. One of them is microbial synergism, which was reported to increase the antibiotic resistance, biofilm development, tissue damage repairing, and environmental adaptation (Dalton et al., 2011; Murray et al., 2014). Previous studies have demonstrated that microbial community composition related to ecosystem functioning constitutes the relationship between phylogeny and functional traits (Cadotte et al., 2008; Gravel et al., 2011). To illustrate the complexity of relationships between different microbial communities, molecular ecological network reconstruction can be considered to represent and model the extent of the complexity (Layeghifard et al., 2016). These reconstructions can be used to explore the characteristics of these polymicrobial interactions (Hacquard et al., 2015).

Diversity in microbial communities could significantly promote stability, productivity, and sustainability in an ecosystem (MacArthur, 1955; Giller et al., 1997). In a steady-state ecosystem, the microbial communities remain stable for a long period of time (Faith et al., 2013), but even a small perturbation (such as antibiotic treatment and/or diet change) can cause considerable changes in the communities (Cho et al., 2012). Obvious succession/shifts of the communities or populations can be detected when their living environments change, especially those subjected to the stresses induced by different organic contaminant. The responses and functional traits of different individuals in a community after an environmental change can be used to predict composition shifts and subsequent effects on the ecosystem (Garnier et al., 2004; Allison and Martiny, 2008; Suding et al., 2008; Díaz et al., 2013). However, phylogenetic changes in different functional bacterial populations with respect to their adaptation processes in the presence of different environmental conditions remain obscure.

In the present study, we aimed to investigate the changes of bacterial communities and their acclimation to the different organic compounds (pyrene and estrogens) as environmental stressors. To this end, phylogenetic analysis and network re-construction of three bacterial phylotypes, affiliated into *Pseudomonadales*, *Vibrionales*, and *Rhodobacterales*, were conducted by examining culturable bacterial communities in cooperation with analyzing the next generation sequencing (NGS) data. We hope the current study would enlighten a better understanding of the bacterial acclimation to the changing environments.

## Materials and Methods

### Field Description and Sampling Procedures

Bacterial strains were isolated from the subsurface sediments collected from the estuary of the Pearl River (Longitude 113.7145 E, Latitude 22.1221 N) at a depth of 8 m during our summer cruise trip (August 15, 2016). The estuary of the Pearl River was selected for sampling due to the abundance of organic pollutants in this region, which has been the result of industrialization and urban development in conjunction with fishing and aquacultural activities in the surrounding areas (Fu et al., 2003). Physiological parameters of the bottom water were measured *in situ*, with a value of 26.5°C for water temperature, 21.6 g/L for salinity, and 7.9 for pH. All samples were kept in 4°C coolers and transferred to the laboratory immediately for further analyses.

### Added Substrates

All organic pollutants used in the present study were of analytical grade or better. A typical PAH pyrene and four different estrogens consisting of estrone (E1), 17β-estradiol (E2), estriol (E3), and 17α-ethinyl estradiol (EE2) were used as environmental stresses (Supplementary Table S1). All of these chemicals were purchased from Sigma–Aldrich, (Shanghai, China) and dissolved in dichloromethane (OceanPAK, Sweden) for preparation of stock solutions. The stock solutions of all organic hydrocarbons and steroids were kept in amber glass vials at -20°C for further use.

### Pyrene and Estrogen Stress Tolerance Assay

The different microbial strains were isolated from the sediment sample collected from the estuary of the Pearl River (Pearl River Estuary). A total of 10 g of sediments were inoculated into 100 mL of mineral salt medium (MSM, 7.01 mM K_2_HPO_4_, 2.94 mM KH_2_PO_4_, 0.81 mM MgSO_4_⋅7H_2_O, 0.18 mM CaCl_2_, 1.71 mM NaCl) (Wang et al., 2016) and incubated in a constant-temperature shaker at 25°C, 150 rpm. After sediment inoculation in MSM, different organic pollutants, comprising 100 mg/L of pyrene and 20 mg/L of E1, E2, E3, and EE2, were added individually as external environmental stresses. Aliquots (100 μL) of liquid cultures at different incubation time points (1, 2, 3, 6, 12, 18, 24, and 30 days) were diluted serially 10-fold and an additional 100 μL of the three dilutions (10^-4^, 10^-5^, and 10^-6^) were spread onto MSM agar plates pretreated with pyrene or the different estrogens (E1, E2, E3, or EE2). The MSM agar plates were prepared by adding 2% of agar powder (BD Biosciences, United States) into MSM liquid medium. All plates were incubated at 25°C for the next 3 days in order to examine microbial growth. The colonies having different morphological features were streaked individually onto MSM agar plates, pre-supplemented with the different organic pollutants, and incubated again for another 3 days at 25°C for growth. A single colony of each isolate was further cultured in marine broth 2216E (BD Biosciences, United States) overnight to cultivate enough bacterial cells for cryopreservation (-80°C with addition of 30% glycerol) and DNA extraction. Genomic DNA of each individual isolate was extracted by using an Ultra-Clean microbial DNA isolation kit (MoBio Laboratories, Carlsbad, CA, United States). The PCR amplification of each isolate’s 16S rRNA gene was carried out using universal primers 27F and 1492R (Wang et al., 2012) and then sequenced at the Beijing Genomics Institute (BGI). The 16S rRNA gene sequence of each isolate was further analyzed by using the BLASTn tool [National Center for Biotechnology Information (NCBI), United States] and presumptively identified on the bases of the top BLAST hits.

To determine the composition of cultured bacterial communities without environmental pressure, marine broth 2216E (BD Biosciences, United States) was used to isolate pure cultures from original sediments using the procedures described above, except that original sediments were enriched in marine broth 2216E for 1 day without the addition of organic pollutants, instead of the MSM medium.

To detect the degrading ability of cultured bacteria with the stress of pyrene, representative bacterial strains were selected different bacterial phylotypes based on phylogenetic analysis. Selected bacterial strains were, respectively, inoculated into 100 mL MSM medium with the supplement of 100mg/L pyrene and incubated in a constant-temperature shaker at 25°C, 150 rpm. Three experimental setups were stopped for culturing at different incubation time points (10, 16, and 21 days, respectively) and used for extract residual pyrene while other experimental setups were kept incubation. Residual pyrene was extracted by dichloromethane analyzed by GC-MS (Agilent 7890-5975c) as the method described before (Wang et al., 2016).

### NGS and Taxonomic Classification

Aliquots (0.25 g) of original sediments and 1.5 ml of liquid cultures at different incubation time points (1, 2, 3, 6, 12, 18, 24, and 30 days) under the stress of pyrene were collected. Genomic DNA was extracted using an Ultra-Clean PowerSoil DNA isolation kit (MoBio Laboratories, Carlsbad, CA, United States). DNA concentrations and purity were measured using a NanoDrop 2000 spectrophotometer (Thermo Fisher Scientific, United States). A total amount of 100 ng (with a concentration of 10 ng/μl) of each sample were sent for sequencing by Illumina HiSeq 2500 platform at the BGI. For the analysis of NGS data, Metaxa2 (Bengtsson-Palme et al., 2015) was used to identify and classify the taxonomies. The 16S rRNA gene sequences of the three functional bacteria groups, *Pseudomonadales*, *Vibrionales*, and *Rhodobacterales*, were selected from all sequencing for the following analysis.

### Clustering of Different Bacterial Phylotypes

Genetic diversity in a specific microbial community can be determined using a common strategy such as the clustering of 16S rRNA sequences into operational taxonomic units (OTUs) based on sequence similarities (Chen et al., 2013). In the present investigation, bacterial isolates were mostly found to be affiliated with three orders (*Pseudomonadales, Vibrionales*, and *Rhodobacterale*s) and the reference sequences downloaded from the NCBI for these three orders were further clustered into different OTUs by using the clustering algorithm, CD-HIT (Li and Godzik, 2006). The selected NGS data was also used CD-HIT to cluster different OTUs. The OTU cutoff value for these sequences used to construct phylogenetic trees was designated as 0.99.

### Analysis of Bacterial Interaction and Community Succession

Bacterial interactions and community succession were analyzed by network-based analytical approaches. These approaches can disentangle complex polymicrobial and microbe–host interactions (Layeghifard et al., 2016). In the present study, the network was constructed using Cytoscape software^1^ (Kohl et al., 2011). Bacterial OTUs clustered by CD-HIT were selected as source interaction elements and different substrates, including pyrene, estrogens, and 2216E, were regarded as target interactive elements when the networks were constructed. Node sizes represented different OTU contributions. Heatmap profiling showed successions of bacterial communities based on OTU presence/absence. Non-metric multidimensional scaling (a multivariate ordination technique, NMDS) based on different OTUs from NGS data was performed to analyze the major shift in structure and composition of bacterial communities.

### Phylogenetic Relevance Analysis

Bacterial isolates belonging to orders *Pseudomonadales*, *Vibrionales*, and *Rhodobacterales* were used to construct phylogenetic trees. These bacterial isolates could account for almost 100% of the isolated bacterial strains. The 16S rRNA gene sequences of strains belonging to these three orders, and reference strains having the highest similarity downloaded from the NCBI database, were then used for phylogenetic tree construction. Neighbor-joining trees were constructed with the Jukes–Cantor correction and bootstrap iteration method (*n* = 1000) (Mishamandani et al., 2016) with reference to *Bacillus* sp. JBS-28, *Acinetobacter venetianus* RMR 1, and *Photobacterium ganghwense* SX1 as outgroups.

## Results

### Composition of Culturable Functional Strains

**FIGURE 1:**
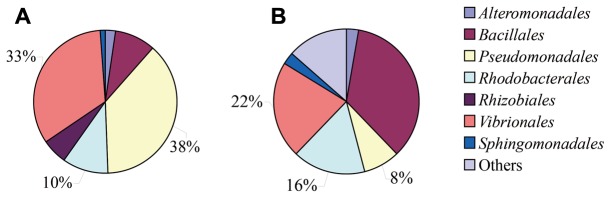
Bacterial community compositions and properties of three orders, *Pseudomonadales*, *Vibrionales*, and *Rhodobacterales*, were listed in the figure. **(A)** pyrene/estrogen-treated samples and **(B)** 2216E-enriched samples.

The sediment samples were added to MSM, and incubated for 30 days with pyrene and estrogens. It was observed that pyrene was almost completely degraded within 30 days of incubation (Supplementary Figure S1). A total of 111 bacterial strains were successfully isolated from MSM media pretreated with pyrene (63 strains at different time intervals over 30 days) and estrogens (48 strains at different time intervals over 30 days, which included 17, 15, 6, and 10 strains from E1, E2, E3, and EE2, respectively). On the other hand, 37 strains were isolated from 2216E medium suspended with sample sediment without the addition of any organic pollutants. A total of 148 strains (63, 48, and 37 from pyrene, estrogen supplemented MSM, and normal 2116E medium, respectively) were characterized by 16S rRNA gene sequencing and aligned with the NCBI website to yield the top BLAST hits corresponding to almost the full length of the 16S rRNA gene sequences. The BLAST results revealed that all of these 148 bacterial isolates were mainly affiliated with seven different orders, including *Pseudomonadales*, *Vibrionales*, *Rhodobacterales*, *Alteromonadales*, *Bacillales*, *Rhizobiales*, and *Sphingomonadales* (**Figure 1**). A large number of significant structural differences among all of the cultured bacterial isolates that were isolated from different experimental setups with the addition of different organic pollutants and normal 2216E medium were observed. The number of bacterial strains belonging to the order *Pseudomonadales* and *Vibrionales* significantly increased under stress conditions (presence of pyrene and estrogens), whereas they were relatively low in proportion comparing to the whole cultured community found in simple 2216E medium (without any organic pollutants). In contrast, bacteria from the *Bacillales* order showed a sharp quantitative decrease under pyrene- and estrogens-treated incubation conditions.

A total of 78 bacterial strains (39, 8, 6, 4, and 4 from pyrene, E1, E2, E3, and EE2 stress environments, respectively, and 17 from normal 2216 E medium) were selected for further investigation in the present study, on the basis of both their potential degradation/toleration capabilities and their large proportions in bacterial communities. These strains were affiliated with three orders, including *Pseudomonadales* (mainly the genus *Pseudomonas* and *Acinetobacter*), *Vibrionales* (mainly the genus *Vibrio* and *Photobacterium*), and *Rhodobacterales*. The 16S rRNA gene sequences of these strains have been deposited in GenBank under accession numbers MF948916–MF948993. These selected bacterial isolates (78 isolates) were further successfully divided into different 37 OTUs using CD-HIT software (cutoff value = 0.99) (Supplementary Table S2). The resulting OTUs were used for diversity, composition, and richness estimations of the whole bacterial community.

Representative bacterial strains based on phylogenetic analysis, including strains PrVl099 and PrVr101, affiliated into the order *Vibrionales*; strains PrPl070 and PrPl084 affiliated into the order *Pseudomonadales*; and strains PrRy136 and PrRr052 affiliated into the order *Rhodobacterales* were used to test the capability of degrading pyrene (Supplementary Figure S2). The results showed that all cultured isolates from the three orders showed great pyrene-degrading capabilities from 31.98 to 43.90%. The bacterium strain PrVl099 was detected to have the greatest degrading efficiency with the value of 43.90%.

### Characterization of Ecological Diversity and Responses to Different Pollutants

**FIGURE 2:**
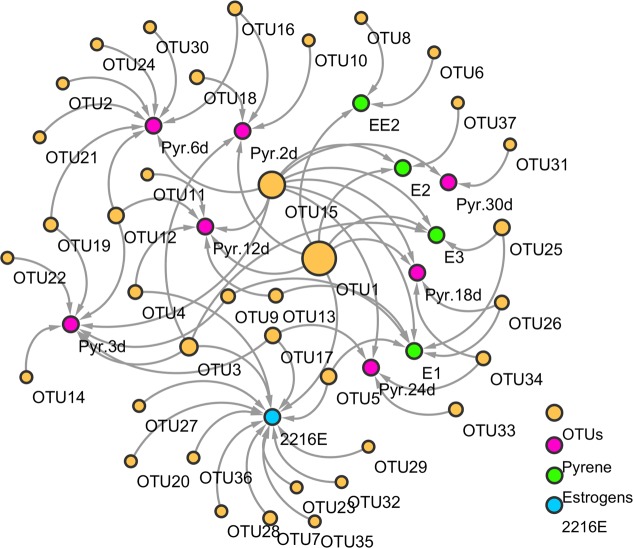
A molecular ecological network construction based on operational taxonomic units (OTUs) generated after treatment with pyrene, estrogens (E1, E2, E3, and EE2), and normal 2216E media. Pink nodes represent the pyrene treatment at different time points, blue nodes represent four different estrogens on day 30 (E1, E2, E3, and EE2), and green nodes represent different bacterial OTUs. Arrow lines between different nodes represent co-occurrences and interactions between different substrates and different bacterial OTUs.

**FIGURE 3:**
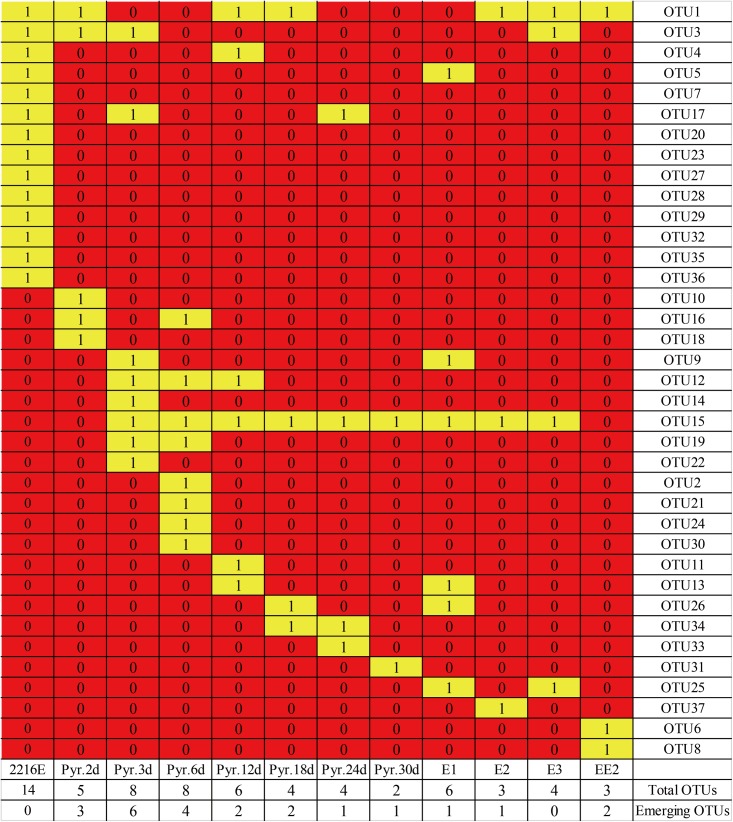
Heatmap profile showing changes in bacterial OTUs after various pollutant-induced stresses (pyrene-treated samples at different time points and four estrogen-treated samples on day 30). Yellow rectangles represent the presence of OTUs in the different substrate treatments.

To illustrate the responses and successions of functional bacterial phylotypes to pyrene- and estrogens-induced stress and non-stress environments (2216E medium), molecular ecological network (**Figure 2**) and heatmap (**Figure 3**) analyses were done.

The correlation interpretation between functional groups of different organic compounds without considering the abundance of different bacterial phylotypes was carried out using molecular ecological network analysis. The pyrene-treated samples appeared to generate more OTUs compared to the estrogen-treated samples. Some OTUs showed an obvious bias to the different substrates. For instance, OTUs 12 and 16 (*Acinetobacter* spp. and *Pseudomonas* spp., respectively) were found only in the pyrene-treated groups, while OTU5 (*Vibrio* spp.) was detected only in the E1-treated samples. Other estrogen-treated (E2) samples also had their own specific OTUs (OTU37, *Donghicola* spp.). Nevertheless, some functional bacterial species also exhibited extensive interactions with different pollutants, which indicated a positive response from these bacterial species toward the different organic pollutants, and hence they might play a significant role in the degradation of different pollutants. For example, OTUs 1 and 15 (*Vibrio* spp. and *Pseudomonas* spp., respectively) were found to be common in both pyrene- and estrogens-treated samples. OTUs 1, 15, and 26 (*Vibrio* spp., *Pseudomonas* spp., and *Labrenzia* spp., respectively) were shared by pyrene- and E1-treated samples. In addition, the OTUs under pyrene tolerance were different from those isolated from MSM plates with added estrogens. For example, OTU15 (*Pseudomonas* spp.) could only be detected after E1 and E3 treatments. The heatmap profile analysis indicated that quantitative changes in different bacterial phylotypes in pyrene- and estrogens-treated samples had occurred. Results from the analysis indicated that some of the functional bacteria emerged in both pyrene- and estrogens-treated groups. The presence of some new bacterial phylotypes was also observed in pyrene-treated samples after 30 days of incubation. Results from the above analysis indicated that, during ecological diversity investigation, certain specific functional phylotypes should also be considered along with whole local communities.

**FIGURE 4:**
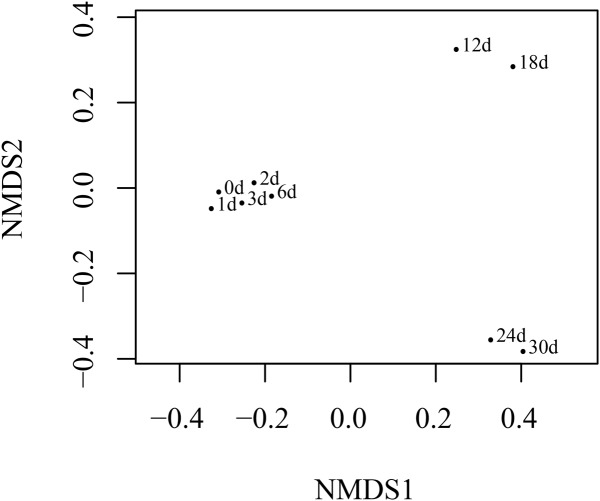
NMDS showing shifts in the quantitative structure of pyrene-degrading communities. Each point was labeled with the corresponding incubation time.

**FIGURE 5:**
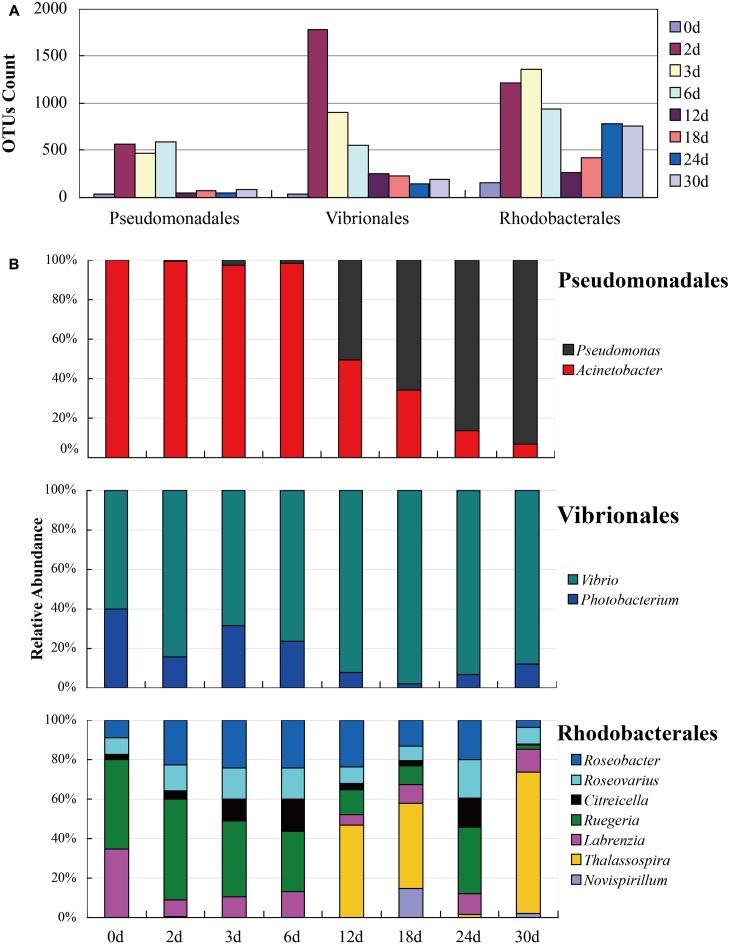
Successions of bacterial communities affiliated into *Pseudomonadales*, *Vibrionales*, and *Rhodobacterales* based on NGS data. **(A)** The OTUs count of functional bacteria; **(B)** The relative abundance of functional bacteria.

The NMDS (**Figure 4**) analysis of the OTUs from NGS data demonstrated that the continuous shift in pyrene-degrading community structure. The NMDS plots showed that relatively large changes in bacterial communities occurred at 12 and 24 days, while small community changes were observed before 6 days. The initial large changes in bacterial community at 12 days could be attributed to adaptation of the seeded bacterial communities to the stress environment. The community profiles from 12 days were most widely dispersed and distantly located from the profiles from the early communities. This seemed to be mainly affected by the maintenance of highly dominant *Alteromonadales* populations and the decrease of *Vibrionales* and *Desulfobacterales* until the end of incubation (Supplementary Figure S3). Metaxa2 (Bengtsson-Palme et al., 2015) was used to select sequences affiliating into orders *Pseudomonadales*, *Vibrionales*, and *Rhodobacterales* from the entire NGS sequencing data. And 2041 OTUs, 8863 OTUs and 5267 OTUs for each bacterial group (Supplementary Table S3) were generated by CD-HIT software. In the presence of pyrene, the number of the OTUs affiliated into *Pseudomonadales*, *Vibrionales*, and *Rhodobacterales* showed dramatical increase at the first 6 days (**Figure 5**). An obvious decrease of OTUs was observed after 12 days for all three bacterial groups which might due to the redundancy reduction. Like composition of culturable bacterial communities, the bacterial communities after 12 days revealed by NGS were also dominated by key functional OTUs, such as OTU P50 (*Acinetobacter* spp.), OTU V0 (*Photobacterium* spp.), and OTU R1 (*Ruegeria* spp.). Moreover, the molecular ecological network based on NGS data (Supplementary Figure S4) showed that the variant density of functional OTUs and the appearance of new phylotypes at different time points.

### Phylogenetic Relevance Analysis

**FIGURE 6:**
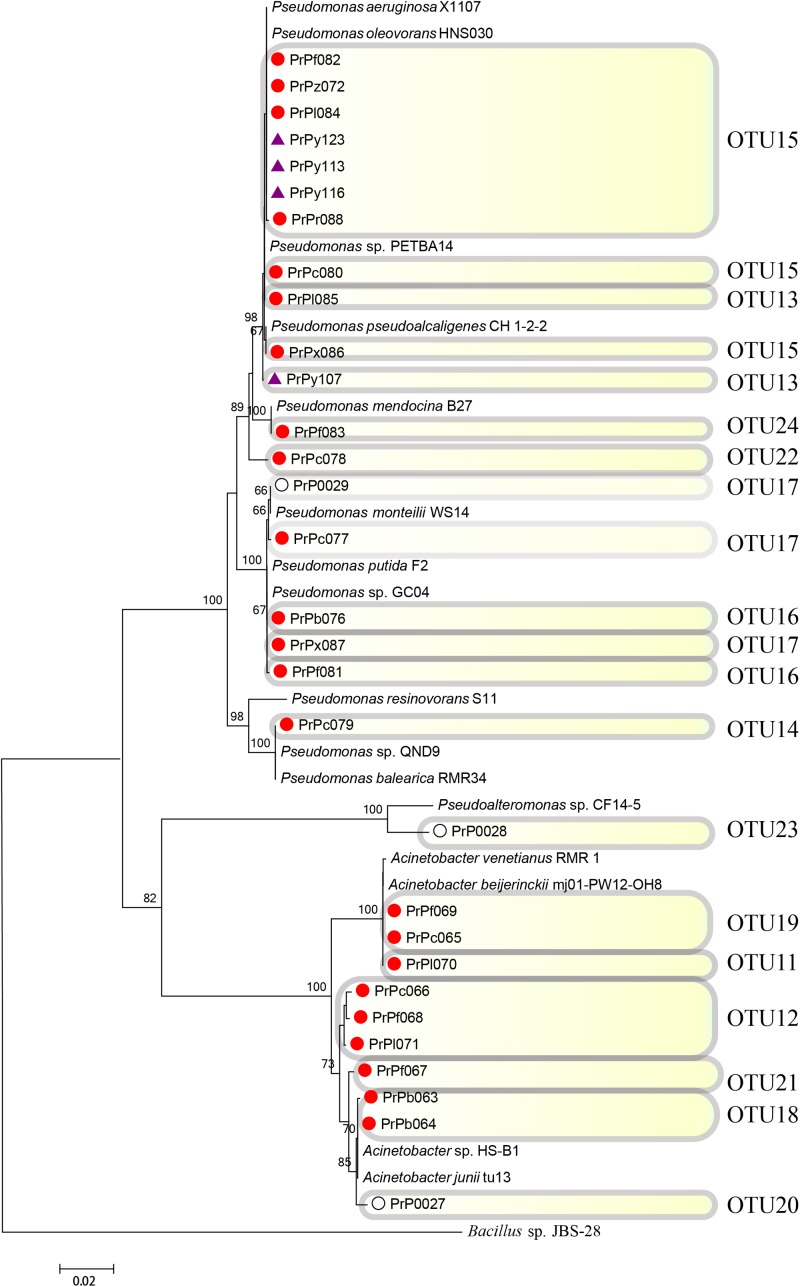
Rooted neighbor-joining phylogenetic tree of partial 16S rRNA gene sequences of bacterial strains (the order *Pseudomonadales*) isolated from estrogen- (purple), pyrene- (red), and 2216E-enriched samples (white) and their corresponding reference strains downloaded from the National Center for Biotechnology Information (NCBI) database. The tree generation and OTU clustering were carried out by MEGA 6 and CD-HIT software, respectively. Bootstrap confidences > 60% were shown at the nodes. *Bacillus* sp. JBS-28 was used as the outgroup.

**FIGURE 7:**
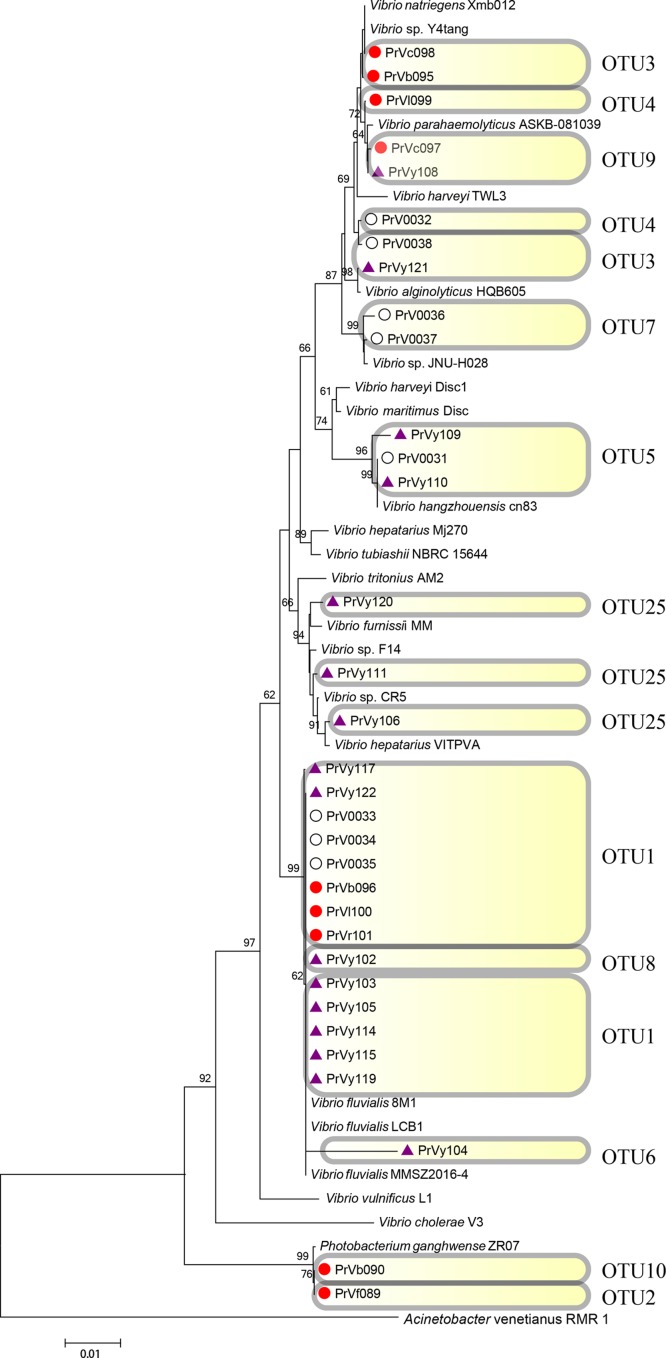
Rooted neighbor-joining phylogenetic tree of partial 16S rRNA gene sequences of bacterial strains (the order *Vibrionales*) isolated from estrogen- (purple), pyrene- (red), and 2216E-pretreated samples (white) and their corresponding reference strains downloaded from NCBI database. The tree generation and OTU clustering were carried out by MEGA 6 and CD-HIT software, respectively. Bootstrap confidences > 60% were shown at the nodes. *Acinetobacter venetianus* RMR 1 was used as the outgroup.

**FIGURE 8:**
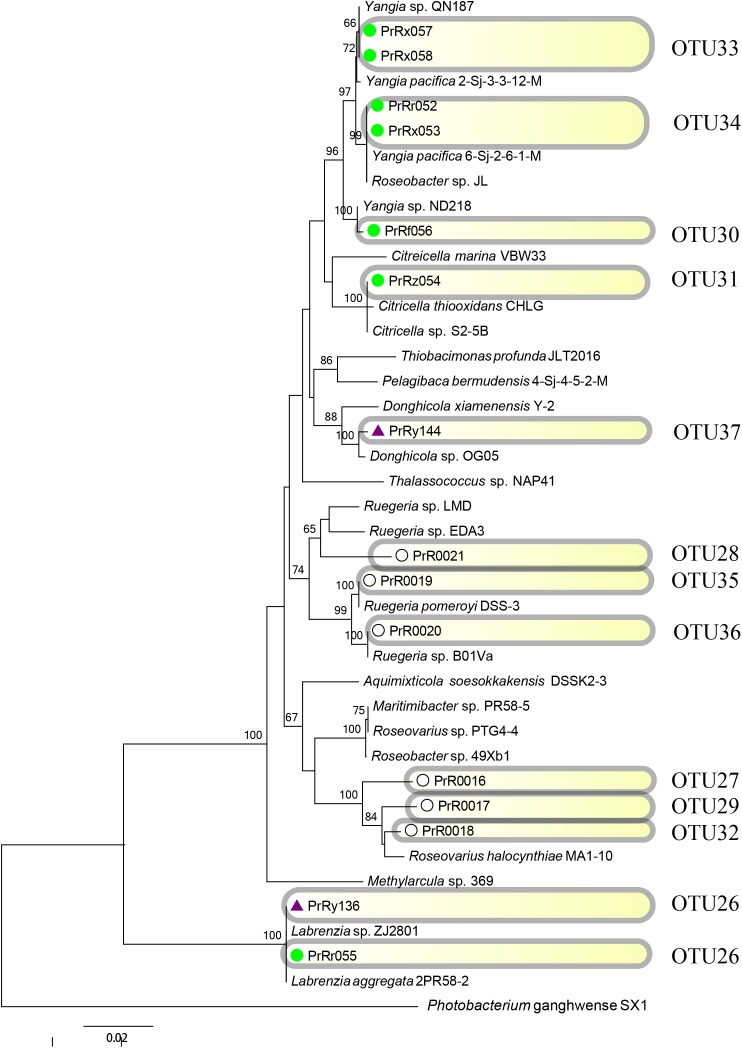
Rooted neighbor-joining phylogenetic tree of partial 16S rRNA gene sequences of bacterial strains (the order *Rhodobacterales*) isolated from estrogen- (purple), pyrene- (green), and 2216E-pretreated samples (white) and their corresponding reference strains downloaded from the NCBI database. The tree generation and OTU clustering were carried out by MEGA 6 and CD-HIT software, respectively. Bootstrap confidences > 60% were shown at the nodes. *Photobacterium ganghwense* SX1 was used as the outgroup.

Comparative phylogenetic studies based on neighbor-joining phylogenetic tree (**Figures 6**–**8** and Supplementary Figure S5) construction were applied to investigate the phylogenetic relevance among different bacterial strains under the stress of different organic contaminants. The 16S rRNA gene sequences (> 1300 bp) amplified from bacterial strains enriched in pyrene- and estrogens-treated culture were used for phylogenetic tree construction.

The phylogenetic tree analysis revealed that the strains PrPc078 and PrPy107 were affiliated with two new OTUs (OTUs 22 and 13) in the genus *Pseudomonas* (**Figure 6**). These strains were regarded as emerging phylotypes, because of their phylogenetic distance from other strains in the tree and their absence in bacterial communities enriched in 2216E medium without any organic pollutant (**Figure 6**). In general, some of the emerged phylotypes were not easily identified from phylogenetic tree analysis, but could be easily discovered with the help of CD-HIT software in the present study. For instance, OTUs 24 and 13, including strains PrPf083 and PrPl085 were identified as emerging taxonomies through the CD-HIT analysis. Based on phylogenetic analysis, certain new phylotypes were also explored in the genus *Acinetobacter*, such as OTU12 (PrPc066, PrPf068, PrPl071), OTU11 (PrPl070), OTU18 (PrPb063 and PrPb064), and OTU21 (PrPf067) (**Figure 6** and Supplementary Figure S5).

Emerging phylotypes were also observed in the tree of the order *Vibrionales* (**Figure 7**); these phylotypes included OTUs 2 and 6 (PrVf089 and PrVy104, respectively). OTUs representing *Photobacterium* spp. (OTUs 2 and 10) and *Vibrio* spp. (OTUs 6 and 8) were detected; these might have been induced by the stress of pyrene and EE2. The order *Rhodobacterales* also exhibited some new phylotype OTUs [for example, OTU30 (PrRf056), and OTU33 (PrRx057 and PrRx058); **Figure 8**]. It was also observed that certain microbial strains belonging to the genus *Yangia* were present in the pyrene-treated incubations but absent in estrogen-treated cultures.

## Discussion

### OTU Profiles in Response to Different Contaminants Stress

In this study, phylogenetic analysis and network re-construction of cultured bacteria communities demonstrated that different organic carbon sources could result in phylogenetic dispersion of functional bacteria. The theory of complex relationships between the genetic diversity of microbial communities, and the functions and stability of microbial systems has been well defined (Tilman, 1996; Girvan et al., 2005). Our experimental results fully supported this theory. The stability of the bacterial community could be negatively influenced by a reduction of genetic diversity and environmental fluctuations. For instance, our results indicated that bacterial species in the order *Rhodobacterales* positively responded to pyrene, but not estrogens. *Rhodobacterales* were acknowledged to be capable of degrading aliphatic and low-molecular-weight aromatic hydrocarbons (Zhang et al., 2004; Harwati et al., 2007). Bacterial strains belonging to the order *Rhodobacterales* were also proved to exhibit less capability for E1 degradation, even at low concentrations (Thayanukul et al., 2010).

An adaptive response was expressed in the microbial strains isolated under the stresses of the organic pollutants. This adaptive feature in certain microbial strains provided them with a superior status in a local ecological environment compared to non-adaptive strains and from being eliminated from the ecosystem under stress conditions (Atlas et al., 1991; Girvan et al., 2005). In our experimental results, OTUs 1 and 15 (*Vibrio* spp. and *Pseudomonas* spp.) were isolated from the pyrene- and the estrogen-treated samples. The sample analysis at different exposure time intervals was considered as a key factor in microbial adaptation to the different organic contaminants. It was well known that bacterial species belonging to the genera *Pseudomonas* and *Vibrio* have efficient capabilities for degrading various organic compounds, including crude oil and estrogens (Kanaly and Harayama, 2000; Yu et al., 2005; Anuar et al., 2013; Lamendella et al., 2014; Burgos-Aceves et al., 2016).

The results also indicated that new phylotypes could emerge in the bacterial community adaptation process in the stress environments. In addition to bacterial strains that originally showed degradation capabilities, the residual cultured community might also mutate in order to adapt to the changing environment, thus generating new phylotypes. The most interesting discovery from the present study was that of OTU functional redundancy accompanied by phylogenetic changes in some bacterial phylotypes. The results of molecular ecological network and heatmap analyses revealed that there were no obvious changes in OTU amounts even when different substrates were used. The presence of new OTUs in samples with the addition of different organic compounds suggests an adaptive response in the surviving groups (Diaz-Ravina and Baath, 1996; Bååth et al., 1998). For instance, the functional population *Vibrio fluvialis* mainly included one phylotype (OTU1) before being co-incubated with EE2, while two new OTUs (OTUs 6 and 8) emerged under EE2-induced stress. Similarly, two new phylotypes (OTUs 13 and 24) affiliated to *Pseudomonas pseudoalcaligenes* were also detected after the addition of the organic pollutants, while only OTU15 was identified in the original samples. These new phylotypes were capable of tolerating or degrading the additional organic pollutants and acted as a substitute for those phylotypes, which were unsuitable to the changing environment, thus led to a community shift. This shift of ecological diversity could be attributed to physiological changes in the community or to the indirect effects on other members of the community (Bååth et al., 1998). Due to the new phylotypes, the ecological diversity of the population and community could remain stable.

Next generation sequencing provided more information of the successions of bacterial communities under the stress of pyrene. Sequences affiliated into the three cultured bacterial orders, *Pseudomonadales* (0.03–3.46%), *Vibrionales* (0.21–26.22%), and *Rhodobacterales* (0.45–4.02%), were abundant and showed dramatic degrading efficiency to pyrene. However, the dominant bacterial sequences at different time points were all affiliated into another bacterial order, *Alteromonadales*. Bacteria in this order were anticipated to have potential function for metabolizing different hydrocarbons (Jin et al., 2012). Considering the dramatic increase of this order, these bacteria were believed to play important roles in degrading pyrene. Unfortunately, bacteria in this order were not successfully isolated in this study. This might ascribe that the majority (> 99%) of microorganisms from the environment was uncultivated in the laboratory (Kaeberlein et al., 2002). In addition, bacteria in the order *Alteromonadales* were often associated to nutrient-rich environments (López-Pérez and Rodriguez-Valera, 2014). However, the refractory organic compound, pyrene, was provided as the sole carbon source in the MSM medium.

### Mechanisms of Bacterial Acclimation

There were several hypotheses to explain the absence or presence of bacterial phylotypes with the treatment of different organic compounds. First, it was due to methodological flaws or primers biases (Donachie et al., 2007). This could be effectively avoided by using pure culture in this study. Second, some “rare” microbes being insensitive to prevailing environmental conditions (Sun et al., 2013) could be selected, which was important in response to the changing environments (organic compounds in this study) (KleinJan et al., 2017). These rare functional bacteria could be neglected by the insufficient sequencing or insufficient culture time (Skopina et al., 2016). These rare bacteria could serve as a “seed bank” (Pedrós-Alió,, 2012), which contributed to the stabilization of bacterial communities (Jousset et al., 2017). Other hypothesis based on current results could be that the occurrence of new phylotypes was caused by evolutionary responses of culturable bacterial communities to the stresses of environmental pollutions. Genetic adaptation provided a means for bacteria adapting or responding to various stresses. The genetic adaptation process comprises three mechanisms: (1) induction and depression of enzymes; (2) genetic changes; and (3) enrichment selection (Leahy and Colwell, 1990).

In the phylogenetic tree analysis of the orders *Pseudomonadales* and *Vibrionales*, novel and long branches were observed, whereas these were absent in the phylogenetic tree of the order *Rhodobacterales* (**Figures 6**–**8**). For instance, these novel branches represent OTUs 12, 18, and 21 in the order *Pseudomonadales* and OTUs 2, 8, and 10 in the order *Vibrionales*, respectively. The evolutionary changes shown in the phylogenetic trees could influence population dynamics, species interactions, and even ecosystem functioning (Post and Palkovacs, 2009; Schoener, 2011; Pfennig and Pfennig, 2012; Matthews et al., 2014). The present results were consistent with previous studies in which wide variations among the strains of *Acinetobacter*, *Vibrio*, and others were illustrated after considering their ability to use different carbon substrates (Sarma et al., 2004; Keymer et al., 2007; Vieira et al., 2011). The role of evolution was well acknowledged in community studies (Weber et al., 2017). The phylogenetic relevance analysis could effectively reveal evolutionary changes among these functional culturable bacteria. Phylogenetic changes were believed to be necessary for the maintenance of the local ecology, especially under extreme environmental conditions. Furthermore, for the relevant comparative phylogenetic studies, there must be proper interplay between ecology and evolution (Weber et al., 2017). The results also indicated that the rapid evolution of functional bacteria responding for changing environments (such as the evolutionary responses of phytoplankton) appeared to be very important for their survival (Padfield et al., 2016).

## Conclusion and Perspectives

In the present communication, organic contaminants (estrogens and pyrene) were used as environmental disturbances or stresses for the analysis of bacterial acclimation. The results revealed that the organic pollutant-induced stresses could significantly influence the bacterial community composition, as well as the acclimation of functional bacteria in these communities. The appearance of new phylotypes under these stress conditions was favorable for community stability as well as for the remediation of the polluted environment. The overall results indicated that the acclimation of functional bacteria was crucial for the adaptation of bacterial communities to environmental disturbances. Considering the finite number of pure cultures and limited information 16S rRNA gene based NGS, this study could not provide a complete picture of the acclimation of bacterial communities to changing environments. The future work could be addressed by (i) analyzing the genomic changes of specific bacterial species to illustrate the mechanism of their capabilities of biodegradation or tolerance; (ii) revealing the co-acclimation and co-evolution of bacterial communities based on metagenomic, metatranscriptomic, and metaproteomic data; and (iii) isolating “uncultivable” functional bacteria in pure culture for comprehensively revealing the function of bacterial community under the press of organic compounds.

## Availability of Data and Materials

All 16S rRNA gene sequences of newly isolated bacterial strains and NGS data are publicly available in the GenBank database (NIH genetic sequence database). The accession numbers for the 16S rRNA gene sequences of cultured bacterial strains are MF948916–MF948993, while the accession numbers for NGS data are listed in Supplementary Table S4.

## Author Contributions

SZ, ZH, and HWa designed the experiment; SZ, XY, JX, CS, and HWe performed the experiment; SZ and HWa collected and analyzed the data and wrote the draft of the article; SZ, AP, ZH, and HWa revised the draft of the article.

## Conflict of Interest Statement

The authors declare that the research was conducted in the absence of any commercial or financial relationships that could be construed as a potential conflict of interest.

## Footnotes

1 http://www.cytoscape.org/

## References

[B1] Allison S. D. Martiny J. B. (2008). Resistance, resilience, and redundancy in microbial communities. *Proc. Natl. Acad. Sci. U.S.A.* 105 11512–11519. 10.1073/pnas.0801925105 18695234 PMC2556421

[B2] Anuar N. M. Kassim M. Sari A. Chan C.-M. (2013). On the bioremediation potential of inhabitant microbes of dredged marine soils: a theoretical framework. *Adv. Mater. Eng. Technol. II* 594–595 173–177. 10.4028/www.scientific.net/KEM.594-595.173

[B3] Atlas R. M. Horowitz A. Krichevsky M. Bej A. K. (1991). Response of microbial populations to environmental disturbance. *Microb. Ecol.* 22 249–256. 10.1007/BF02540227 24194340

[B4] Bååth E. Díaz-Raviña M. Frostegård Å. Campbell C. D. (1998). Effect of metal-rich sludge amendments on the soil microbial community. *Appl. Environ. Microbiol.* 64 238–245. 16349483 10.1128/aem.64.1.238-245.1998 PMC124700

[B5] Bengtsson-Palme J. Hartmann M. Eriksson K. M. Pal C. Thorell K. Larsson D. G. J. (2015). METAXA2: improved identification and taxonomic classification of small and large subunit rRNA in metagenomic data. *Mol. Ecol. Resour.* 15 1403–1414. 10.1111/1755-0998.12399 25732605

[B6] Burgos-Aceves M. A. Cohen A. Smith Y. Faggio C. (2016). Estrogen regulation of gene expression in the teleost fish immune system. *Fish Shellfish Immunol.* 58 42–49. 10.1016/j.fsi.2016.09.006 27633675

[B7] Cadotte M. W. Cardinale B. J. Oakley T. H. (2008). Evolutionary history and the effect of biodiversity on plant productivity. *Proc. Natl. Acad. Sci. U.S.A.* 105 17012–17017. 10.1073/pnas.0805962105 18971334 PMC2579369

[B8] Chen W. Zhang C. K. Cheng Y. Zhang S. Zhao H. (2013). A comparison of methods for clustering 16S rRNA sequences into OTUs. *PLoS One* 8:e70837. 10.1371/journal.pone.0070837 23967117 PMC3742672

[B9] Cho I. Yamanishi S. Cox L. Methé B. A. Zavadil J. Li K. (2012). Antibiotics in early life alter the murine colonic microbiome and adiposity. *Nature* 488 621–626. 10.1038/nature11400 22914093 PMC3553221

[B10] da Silva D. P. Castañeda-Ojeda M. P. Moretti C. Buonaurio R. Ramos C. Venturi V. (2014). Bacterial multispecies studies and microbiome analysis of a plant disease. *Microbiology* 160 556–566. 10.1099/mic.0.074468-0 24421406

[B11] Dalton T. Dowd S. E. Wolcott R. D. Sun Y. Watters C. Griswold J. A. (2011). An *in vivo* polymicrobial biofilm wound infection model to study interspecies interactions. *PLoS One* 6:e27317. 10.1371/journal.pone.0027317 22076151 PMC3208625

[B12] Dash H. R. Mangwani N. Chakraborty J. Kumari S. Das S. (2013). Marine bacteria: potential candidates for enhanced bioremediation. *Appl. Microbiol. Biotechnol.* 97 561–571. 10.1007/s00253-012-4584-0 23212672

[B13] Díaz S. Purvis A. Cornelissen J. H. Mace G. M. Donoghue M. J. Ewers R. M. (2013). Functional traits, the phylogeny of function, and ecosystem service vulnerability. *Ecol. Evol.* 3 2958–2975. 10.1002/ece3.601 24101986 PMC3790543

[B14] Diaz-Ravina M. Baath E. (1996). Development of metal tolerance in soil bacterial communities exposed to experimentally increased metal levels. *Appl. Environ. Microbiol.* 62 2970–2977. 16535383 10.1128/aem.62.8.2970-2977.1996 PMC1388921

[B15] Donachie S. P. Foster J. S. Brown M. V. (2007). Culture clash: challenging the dogma of microbial diversity. *ISME J.* 1 97–99. 10.1038/ismej.2007.22 18043618

[B16] Faith J. J. Guruge J. L. Charbonneau M. Subramanian S. Seedorf H. Goodman A. L. (2013). The long-term stability of the human gut microbiota. *Science* 341:1237439. 10.1126/science.1237439 23828941 PMC3791589

[B17] Fu J. Mai B. Sheng G. Zhang G. Wang X. Peng P. A. (2003). Persistent organic pollutants in environment of the Pearl River Delta, China: an overview. *Chemosphere* 52 1411–1422. 10.1016/S0045-6535(03)00477-6 12867171

[B18] Garnier E. Cortez J. Billès G. Navas M.-L. Roumet C. Debussche M. (2004). Plant functional markers capture ecosystem properties during secondary succession. *Ecology* 85 2630–2637. 10.1890/03-0799

[B19] Giller K. Beare M. Lavelle P. Izac A.-M. Swift M. (1997). Agricultural intensification, soil biodiversity and agroecosystem function. *Appl. Soil Ecol.* 6 3–16. 10.1016/S0929-1393(96)00149-7

[B20] Girvan M. Campbell C. Killham K. Prosser J. Glover L. (2005). Bacterial diversity promotes community stability and functional resilience after perturbation. *Environ. Microbiol.* 7 301–313. 10.1111/j.1462-2920.2005.00695.x 15683391

[B21] Gravel D. Bell T. Barbera C. Bouvier T. Pommier T. Venail P. (2011). Experimental niche evolution alters the strength of the diversity-productivity relationship. *Nature* 469 89–92. 10.1038/nature09592 21131946

[B22] Hacquard S. Garrido-Oter R. González A. Spaepen S. Ackermann G. Lebeis S. (2015). Microbiota and host nutrition across plant and animal kingdoms. *Cell Host Microbe* 17 603–616. 10.1016/j.chom.2015.04.009 25974302

[B23] Hansen T. H. Gøbel R. J. Hansen T. Pedersen O. (2015). The gut microbiome in cardio-metabolic health. *Genome Med.* 7:33. 10.1186/s13073-015-0157-z 25825594 PMC4378584

[B24] Haritash A. Kaushik C. (2009). Biodegradation aspects of polycyclic aromatic hydrocarbons (PAHs): a review. *J. Hazard. Mater.* 169 1–15. 10.1016/j.jhazmat.2009.03.137 19442441

[B25] Harwati T. U. Kasai Y. Kodama Y. Susilaningsih D. Watanabe K. (2007). Characterization of diverse hydrocarbon-degrading bacteria isolated from Indonesian seawater. *Microb. Environ.* 22 412–415. 10.1264/jsme2.22.412

[B26] Jin H. M. Kim J. M. Lee H. J. Madsen E. L. Jeon C. O. (2012). Alteromonas as a key agent of polycyclic aromatic hydrocarbon biodegradation in crude oil-contaminated coastal sediment. *Environ. Sci. Technol.* 46 7731–7740. 10.1021/es3018545 22709320

[B27] Jousset A. Bienhold C. Chatzinotas A. Gallien L. Gobet A. Kurm V. (2017). Where less may be more: how the rare biosphere pulls ecosystems strings. *ISME J.* 11 853–862. 10.1038/ismej.2016.174 28072420 PMC5364357

[B28] Kaeberlein T. Lewis K. Epstein S. S. (2002). Isolating “uncultivable” microorganisms in pure culture in a simulated natural environment. *Science* 296 1127–1129. 10.1126/science.1070633 12004133

[B29] Kanaly R. A. Harayama S. (2000). Biodegradation of high-molecular-weight polycyclic aromatic hydrocarbons by bacteria. *J. Bacteriol.* 182 2059–2067. 10.1128/JB.182.8.2059-2067.2000 10735846 PMC111252

[B30] Keymer D. P. Miller M. C. Schoolnik G. K. Boehm A. B. (2007). Genomic and phenotypic diversity of coastal *Vibrio cholerae* strains is linked to environmental factors. *Appl. Environ. Microbiol.* 73 3705–3714. 10.1128/AEM.02736-06 17449702 PMC1932678

[B31] KleinJan H. Jeanthon C. Boyen C. Dittami S. M. (2017). Exploring the cultivable *Ectocarpus* microbiome. *Front. Microbiol.* 8:2456 10.3389/fmicb.2017.02456 PMC5732352 29312170

[B32] Kohl M. Wiese S. Warscheid B. (2011). Cytoscape: software for visualization and analysis of biological networks. *Methods Mol. Biol.* 696 291–303. 10.1007/978-1-60761-987-1_18 21063955

[B33] Lamendella R. Strutt S. Borglin S. Chakraborty R. Tas N. Mason O. U. (2014). Assessment of the Deepwater Horizon oil spill impact on Gulf coast microbial communities. *Front. Microbiol.* 5:130. 10.3389/fmicb.2014.00130 24772107 PMC3982105

[B34] Layeghifard M. Hwang D. M. Guttman D. S. (2016). Disentangling interactions in the microbiome: a network perspective. *Trends Microbiol.* 25 217–228. 10.1016/j.tim.2016.11.008 27916383 PMC7172547

[B35] Leahy J. G. Colwell R. R. (1990). Microbial degradation of hydrocarbons in the environment. *Microbiol. Rev.* 54 305–315. 2215423 10.1128/mr.54.3.305-315.1990 PMC372779

[B36] Li W. Godzik A. (2006). Cd-hit: a fast program for clustering and comparing large sets of protein or nucleotide sequences. *Bioinformatics* 22 1658–1659. 10.1093/bioinformatics/btl158 16731699

[B37] López-Pérez M. Rodriguez-Valera F. (2014). “The family *Alteromonadaceae*,” in *The Prokaryotes*, eds Rosenberg E. DeLong E. F. Lory S. Stackebrandt E. Thompson F. (Berlin: Springer), 69–92. 10.1007/978-3-642-38922-1_233

[B38] MacArthur R. (1955). Fluctuations of animal populations and a measure of community stability. *Ecology* 36 533–536. 10.2307/1929601

[B39] Matthews B. De Meester L. Jones C. G. Ibelings B. W. Bouma T. J. Nuutinen V. (2014). Under niche construction: an operational bridge between ecology, evolution, and ecosystem science. *Ecol. Monogr.* 84 245–263. 10.1890/13-0953.1

[B40] McDevitt-Irwin J. Baum J. Garren M. Vega Thurber R. (2017). Responses of coral-associated bacterial communities to local and global stressors. *Front. Mar. Sci.* 4:262 10.3389/fmars.2017.00262

[B41] Mishamandani S. Gutierrez T. Berry D. Aitken M. D. (2016). Response of the bacterial community associated with a cosmopolitan marine diatom to crude oil shows a preference for the biodegradation of aromatic hydrocarbons. *Environ. Microbiol.* 18 1817–1833. 10.1111/1462-2920.12988 26184578 PMC4970210

[B42] Murray J. L. Connell J. L. Stacy A. Turner K. H. Whiteley M. (2014). Mechanisms of synergy in polymicrobial infections. *J. Microbiol.* 52 188–199. 10.1007/s12275-014-4067-3 24585050 PMC7090983

[B43] Padfield D. Yvon-Durocher G. Buckling A. Jennings S. Yvon-Durocher G. (2016). Rapid evolution of metabolic traits explains thermal adaptation in phytoplankton. *Ecol. Lett.* 19 133–142. 10.1111/ele.12545 26610058 PMC4991271

[B44] Pedrós-Alió C. (2012). The rare bacterial biosphere. *Annu. Rev. Mar. Sci.* 4 449–466. 10.1146/annurev-marine-120710-100948 22457983

[B45] Pfennig D. W. Pfennig K. S. (2012). Evolution’s wedge: competition and the origins of diversity. *Bioscience* 63 770–771. 10.1525/bio.2013.63.9.15

[B46] Post D. M. Palkovacs E. P. (2009). Eco-evolutionary feedbacks in community and ecosystem ecology: interactions between the ecological theatre and the evolutionary play. *Philos. Trans. R. Soc. Lond. Ser. B* 364 1629–1640. 10.1098/rstb.2009.0012 19414476 PMC2690506

[B47] Rogers G. B. Hoffman L. R. Carroll M. P. Bruce K. D. (2013). Interpreting infective microbiota: the importance of an ecological perspective. *Trends Microbiol.* 21 271–276. 10.1016/j.tim.2013.03.004 23598051 PMC3880558

[B48] Sarma P. M. Bhattacharya D. Krishnan S. Lal B. (2004). Assessment of intra-species diversity among strains of *Acinetobacter baumannii* isolated from sites contaminated with petroleum hydrocarbons. *Can. J. Microbiol.* 50 405–414. 10.1139/w04-018 15284886

[B49] Schoener T. W. (2011). The newest synthesis: understanding the interplay of evolutionary and ecological dynamics. *Science* 331 426–429. 10.1126/science.1193954 21273479

[B50] Skopina M. Vasileva A. Pershina E. Pinevich A. (2016). Diversity at low abundance: the phenomenon of the rare bacterial biosphere. *Microbiology* 85 272–282. 10.1134/S0026261716030139

[B51] Stecher B. Denzler R. Maier L. Bernet F. Sanders M. J. Pickard D. J. (2012). Gut inflammation can boost horizontal gene transfer between pathogenic and commensal *Enterobacteriaceae*. *Proc. Natl. Acad. Sci. U.S.A.* 109 1269–1274. 10.1073/pnas.1113246109 22232693 PMC3268327

[B52] Suding K. N. Lavorel S. Chapin F. Cornelissen J. H. Diaz S. Garnier E. (2008). Scaling environmental change through the community-level: a trait-based response-and-effect framework for plants. *Glob. Change Biol.* 14 1125–1140. 10.1111/j.1365-2486.2008.01557.x

[B53] Sun M. Y. Dafforn K. A. Johnston E. L. Brown M. V. (2013). Core sediment bacteria drive community response to anthropogenic contamination over multiple environmental gradients. *Environ. Microbiol.* 15 2517–2531. 10.1111/1462-2920.12133 23647974

[B54] Thayanukul P. Zang K. Janhom T. Kurisu F. Kasuga I. Furumai H. (2010). Concentration-dependent response of estrone-degrading bacterial community in activated sludge analyzed by microautoradiography-fluorescence in situ hybridization. *Water Res.* 44 4878–4887. 10.1016/j.watres.2010.07.031 20705312

[B55] Tilman D. (1996). Biodiversity: population versus ecosystem stability. *Ecology* 77 350–363. 10.2307/2265614

[B56] Vieira G. Sabarly V. Bourguignon P.-Y. Durot M. Le Fèvre F. Mornico D. (2011). Core and panmetabolism in *Escherichia coli*. *J. Bacteriol.* 193 1461–1472. 10.1128/JB.01192-10 21239590 PMC3067614

[B57] Wang H. Laughinghouse H. D. Anderson M. A. Chen F. Willliams E. Place A. R. (2012). Novel bacterial isolate from Permian groundwater, capable of aggregating potential biofuel-producing microalga *Nannochloropsis oceanica* IMET1. *Appl. Environ. Microbiol.* 78 1445–1453. 10.1128/aem.06474-11 22194289 PMC3294481

[B58] Wang H. Wang B. Dong W. W. Hu X. K. (2016). Co-acclimation of bacterial communities under stresses of hydrocarbons with different structures. *Sci. Rep.* 6:34588. 10.1038/srep34588 27698451 PMC5048299

[B59] Weber M. G. Wagner C. E. Best R. J. Harmon L. J. Matthews B. (2017). Evolution in a community context: on integrating ecological interactions and macroevolution. *Trends Ecol. Evol.* 32 291–304. 10.1016/j.tree.2017.01.003 28215448

[B60] Yu C.-P. Ahuja R. Sayler G. Chu K.-H. (2005). Quantitative molecular assay for fingerprinting microbial communities of wastewater and estrogen-degrading consortia. *Appl. Environ. Microbiol.* 71 1433–1444. 10.1128/AEM.71.3.1433-1444.2005 15746346 PMC1065124

[B61] Zhang H. Kallimanis A. Koukkou A. I. Drainas C. (2004). Isolation and characterization of novel bacteria degrading polycyclic aromatic hydrocarbons from polluted Greek soils. *Appl. Microbiol. Biotechnol.* 65 124–131. 10.1007/s00253-004-1614-6 15133642

