# Supplementary material for: Acclimation of Culturable Bacterial Communities under the Stresses of Different Organic Compounds
Source: Front Microbiol. 2018 Feb 19;9:225. doi: 10.3389/fmicb.2018.00225 (PMC5827545; doi:10.3389/fmicb.2018.00225)
Supplement: Supplementary file 1 [file Data_Sheet_1.DOC]

**Acclimation of Bacterial Communities under the Stresses of Different Organic Compounds**

Hui Wang^#^, Shuangfei Zhang^#^, Amit Pratush, Xueying Ye, Jinli Xie, Huan Wei, Chongran Sun, Zhong Hu^*^

Biology Department, College of Science, Shantou University, Shantou, China 515063

Corresponding author: Zhong Hu, hzh@stu.edu.cn, +86-754-86504189

# These two authors contribute equally to this work.


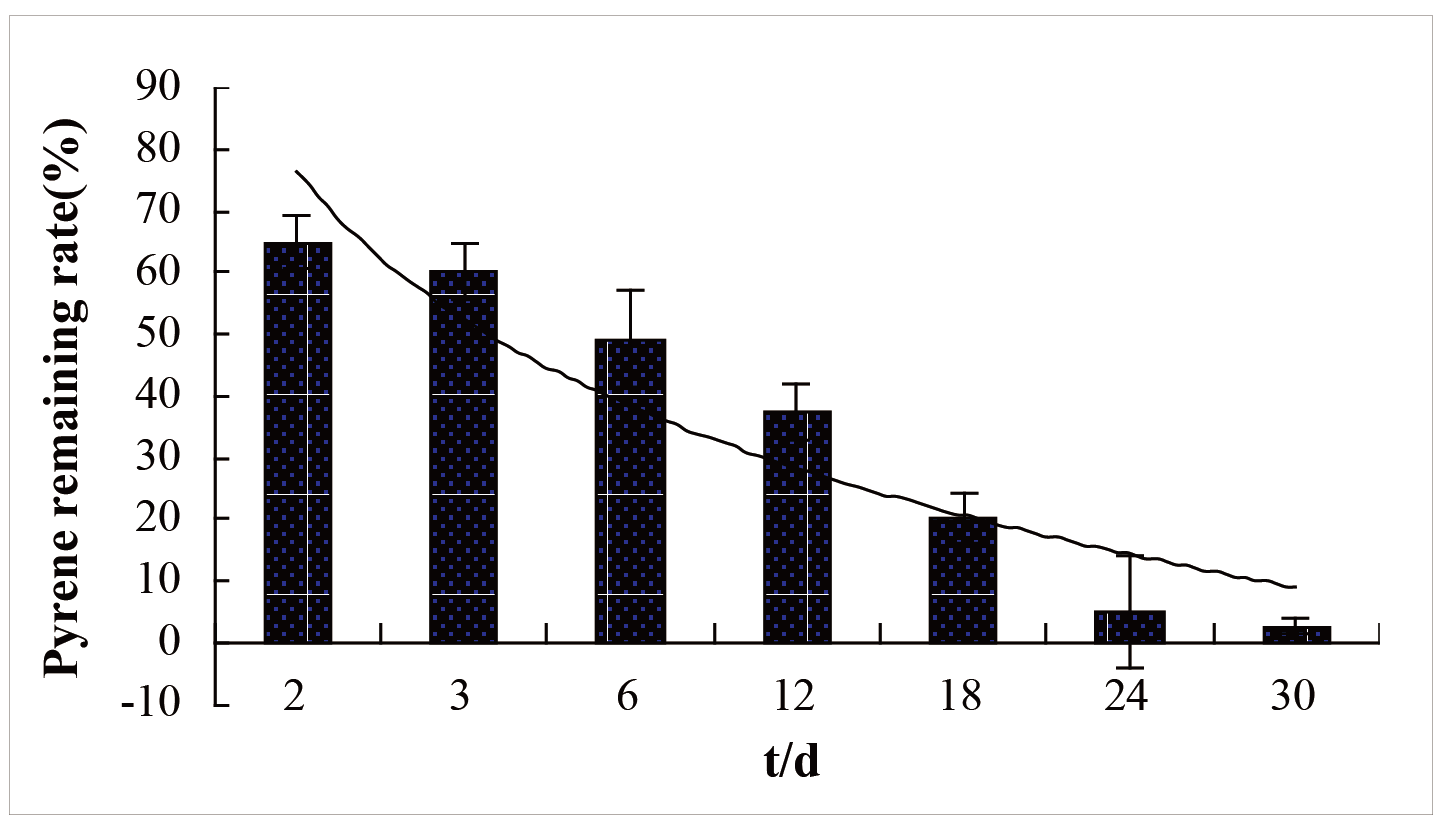


SUPPLEMENTARY FIGURE S1 | Degradation curve of pyrene for 30 days.


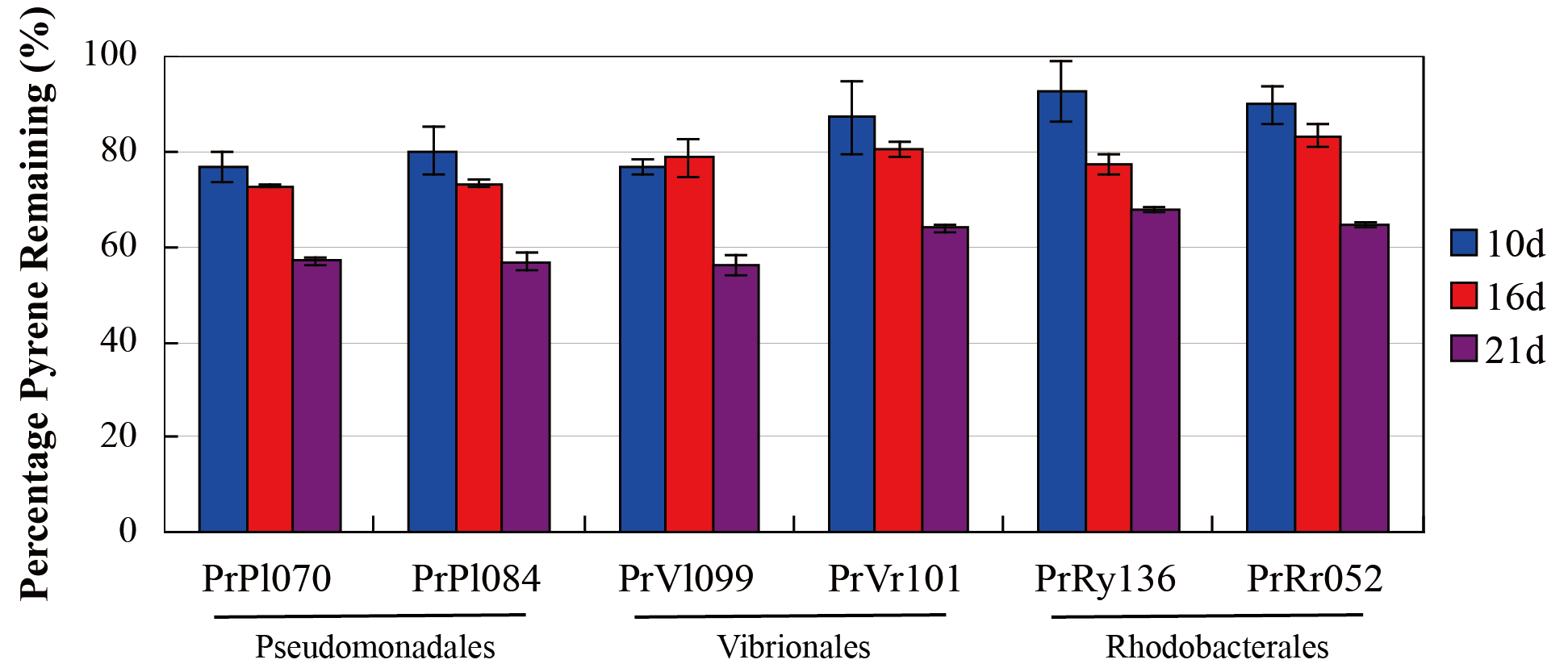


SUPPLEMENTARY FIGURE S2 | Percentage pyrene remaining rates of six strains.


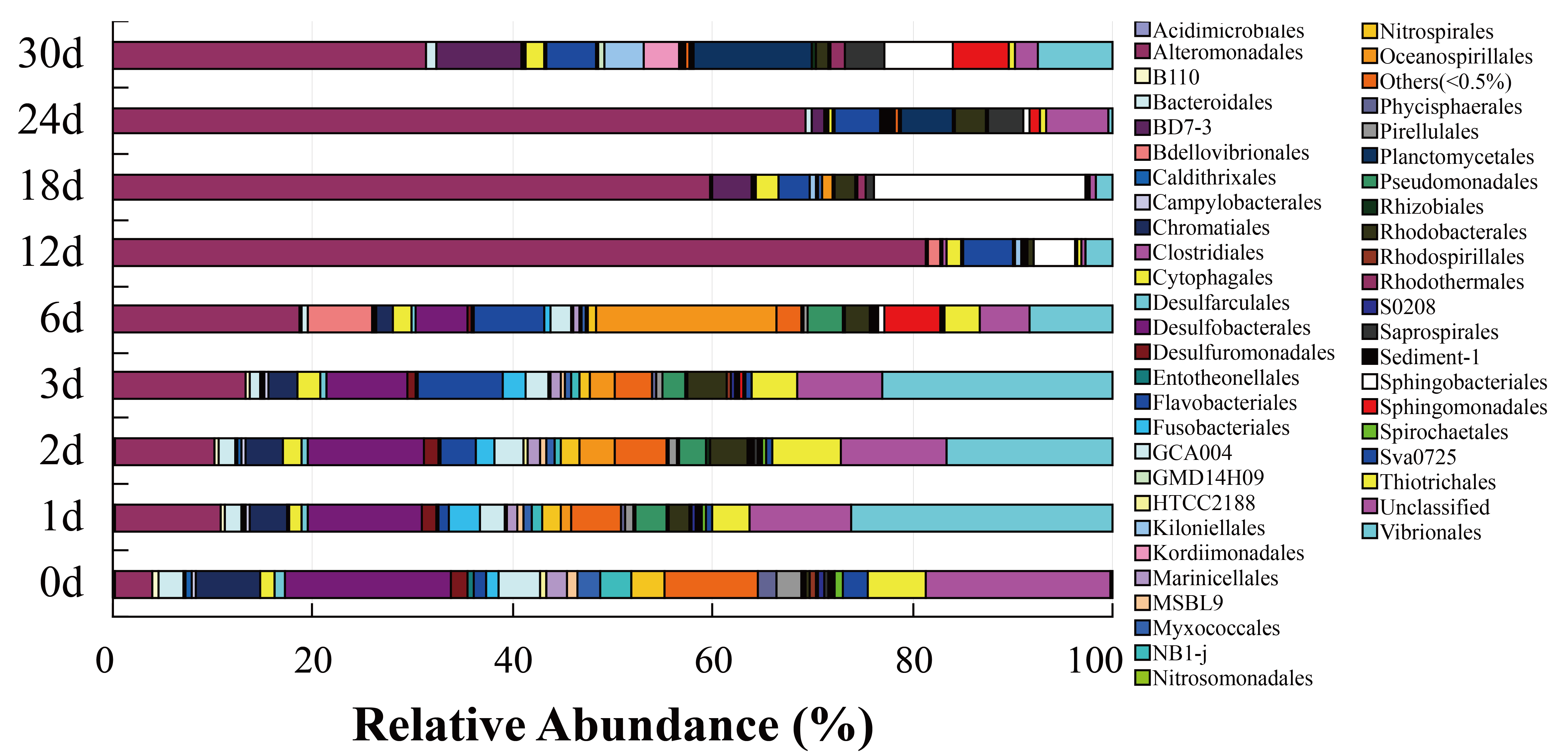


SUPPLEMENTARY FIGURE S3 | Relative abundance of different pyrene-degrading communities over treatment time (Family level).


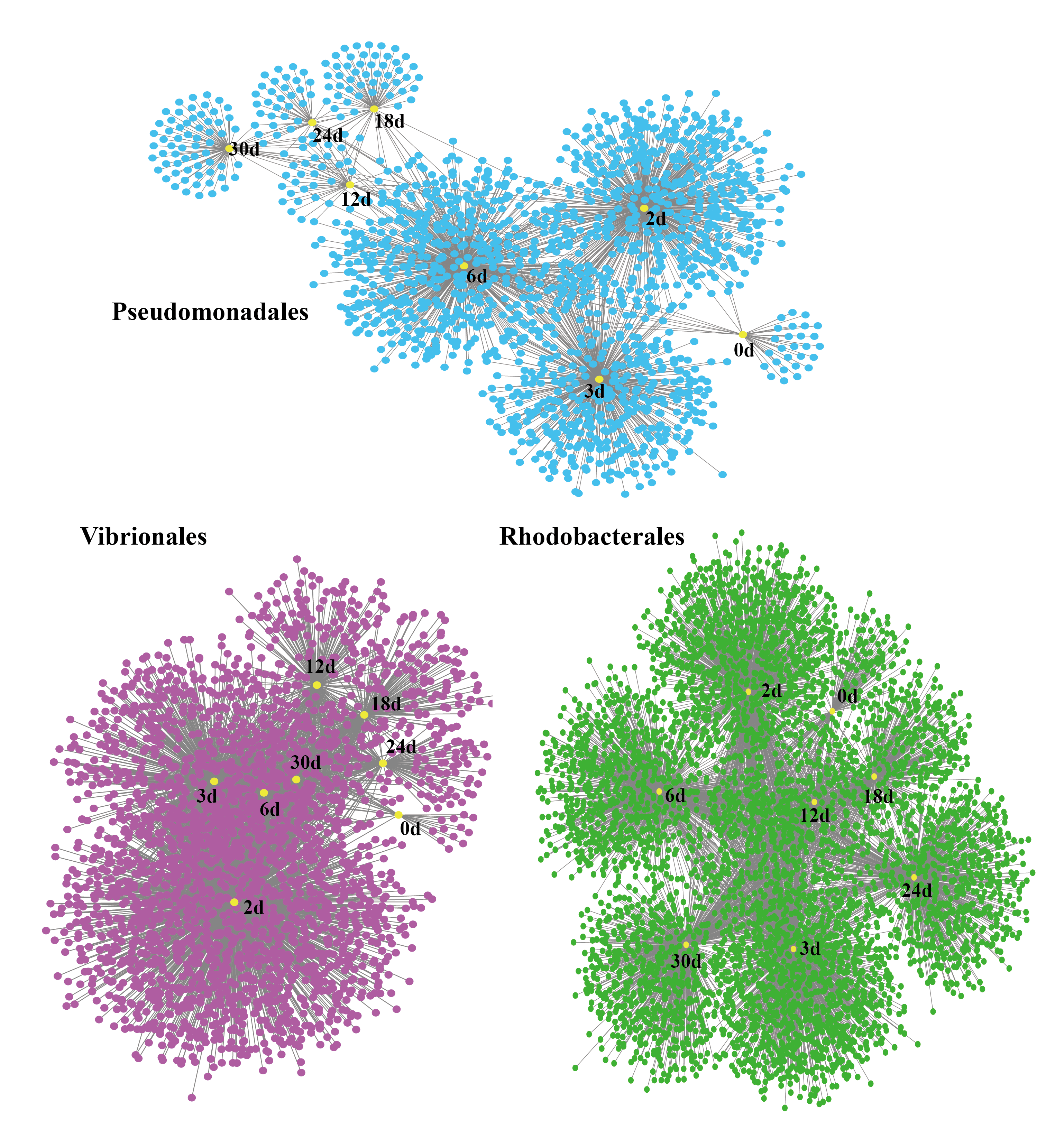


SUPPLEMENTARY FIGURE S4 |A molecular ecological network construction based on NGS data generated after treatment with pyrene. Yellow nodes represent the pyrene treatment at different time points, other nodes represent different bacterial OTUs. Arrow lines between different nodes represent co-occurrences and interactions between bacteria and different bacterial OTUs.


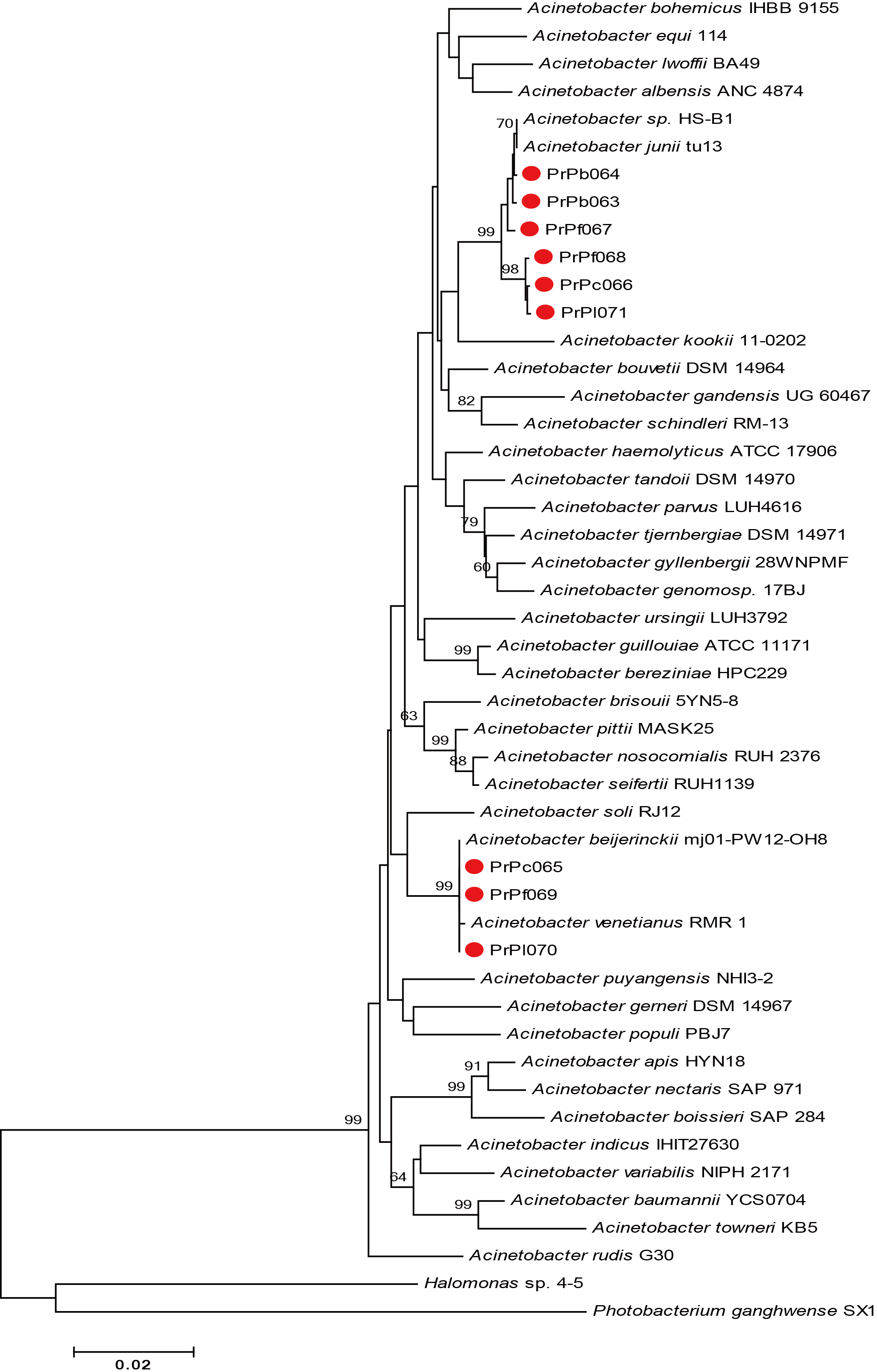


SUPPLEMENTARY FIGURE S5 |Rooted neighbor-joining tree of partial 16S rRNA gene sequences of bacterial (the genus *Acinetobacter*) strains isolated from pyrene treated samples and their corresponding reference strains download from NCBI database. The tree was generated by MEGA 6 software. GenBank accession number were listed after each sequence. Bootstrap confidence greater than 60% were shown at the nodes. *Halomonas* sp. 4-5 and *Photobacterium ganghwense* SX1 were applied here as the outgroup.SUPPLEMENTARY TABLE S1 | The information summit of added organic pollutants.

| Added Substrates | Abbreviation | Final concentration (mg/L) | Category |
| --- | --- | --- | --- |
| Pyrene | Pyr | 100 | PAHs |
| Estrone | E1 | 20 | Estorgens |
| 17β-estradiol | E2 | 20 | Estorgens |
| Estriol | E3 | 20 | Estorgens |
| 17α-ethinyl estradiol | EE2 | 20 | Estorgens |

SUPPLEMENTARY TABLE S2 | OTUs defined by 16S rRNA gene sequences (Cutoff Value = 0.99).

| **OTUs** | **Size** | **Strains** | **Substrates** | **Accessions** | **Similarity** | **Closed-Relative strains from NCBI** |
| --- | --- | --- | --- | --- | --- | --- |
| **OTU1** |  |  |  |  |  |  |
| 0 | 1461nt | PrVl100 | Pyr.12d | MF948990 | * | *Vibrio fluvialis* LCB1 |
| 1 | 1454nt | PrVy115 | E2 | MF948970 | 99.38% |
| 2 | 1453nt | PrVy117 | E2 | MF948971 | 99.45% |
| 3 | 1452nt | PrVy119 | E2 | MF948972 | 99.45% |
| 4 | 1455nt | PrV0033 | 2216E | MF948965 | 99.38% | *Vibrio fluvialis* EP27-13-11 |
| 5 | 1454nt | PrV0034 | 2216E | MF948966 | 99.31% |
| 6 | 1456nt | PrV0035 | 2216E | MF948967 | 99.24% |
| 7 | 1454nt | PrVb096 | Pyr.2d | MF948987 | 99.52% |
| 8 | 1453nt | PrVr101 | Pyr.18d | MF948991 | 99.31% |
| 9 | 1455nt | PrVy103 | EE2 | MF948973 | 99.11% | *Vibrio fluvialis* MMSZ2016-4 |
| 10 | 1452nt | PrVy114 | EE2 | MF948969 | 99.66% |
| 11 | 1414nt | PrVy105 | EE2 | MF948975 | 100.00% | *Vibrio fluvialis* 8M1 |
| 12 | 1387nt | PrVy122 | E3 | MF948979 | 100.00% |
| **OTU2** |  |  |  |  |  |  |
| 0 | 1460nt | PrVf089 | Pyr.6d | MF948993 | * | *Photobacterium ganghwense* ZR07 |
| **OTU3** |  |  |  |  |  |  |
| 0 | 1452nt | PrV0038 | 2216E | MF948968 | 99.52% | *Vibrio* sp. Y4tang |
| 1 | 1455nt | PrVb095 | Pyr.2d | MF948985 | * | *Vibrio natriegens* Xmb012 |
| 2 | 1450nt | PrVc098 | Pyr.3d | MF948988 | 99.31% | *Vibrio azureus* Xmb005 |
| 3 | 1412nt | PrVy121 | E3 | MF948978 | 99.29% | *Vibrio alginolyticus* HQB605 |
| **OTU4** |  |  |  |  |  |  |
| 0 | 1451nt | PrV0032 | 2216E | MF948961 | 99.52% | *Vibrio* sp. Y4tang |
| 1 | 1455nt | PrVl099 | Pyr.12d | MF948989 | * | *Vibrio azureus* CAIM 1457 |
| **OTU5** |  |  |  |  |  |  |
| 0 | 1454nt | PrV0031 | 2216E | MF948964 | * | *Vibrio hangzhouensis* cn83 |
| 1 | 1417nt | PrVy109 | E1 | MF948981 | 99.36% |
| 2 | 1419nt | PrVy110 | E1 | MF948982 | 99.93% |
| **OTU6** |  |  |  |  |  |  |
| 0 | 1454nt | PrVy104 | EE2 | MF948974 | * | *Vibrio fluvialis* MMSZ2016-4 |
| **OTU7** |  |  |  |  |  |  |
| 0 | 1451nt | PrV0036 | 2216E | MF948963 | 99.24% | *Vibrio natriegens* Xmb012 |
| 1 | 1453nt | PrV0037 | 2216E | MF948962 | * | *Vibrio* sp. JNU-H028 |
| **OTU8** |  |  |  |  |  |  |
| 0 | 1453nt | PrVy102 | EE2 | MF948984 | * | *Vibrio fluvialis* MMSZ2016-4 |
| **OTU9** |  |  |  |  |  |  |
| 0 | 1452nt | PrVc097 | Pyr.3d | MF948992 | * | *Vibrio* sp. M-137-19 |
| 1 | 1416nt | PrVy108 | E1 | MF948980 | 99.72% | *Vibrio parahaemolyticus* CHN-6 |
| **OTU10** |  |  |  |  |  |  |
| 0 | 1451nt | PrVb090 | Pyr.2d | MF948986 | * | *Photobacterium ganghwense* SX1 |
| **OTU11** |  |  |  |  |  |  |
| 0 | 1449nt | PrPl070 | Pyr.12d | MF948927 | * | *Acinetobacter beijerinckii* mj01-PW12-OH8 |
| **OTU12** |  |  |  |  |  |  |
| 0 | 1438nt | PrPc066 | Pyr.3d | MF948942 | 99.30% | *Acinetobacter* sp. HS-B1 |
| 1 | 1434nt | PrPf068 | Pyr.6d | MF948944 | 99.23% |
| 2 | 1447nt | PrPl071 | Pyr.12d | MF948945 | * |
| **OTU13** |  |  |  |  |  |  |
| 0 | 1447nt | PrPl085 | Pyr.12d | MF948928 | * | *Pseudomonas pseudoalcaligenes* RMR17 |
| 1 | 1402nt | PrPy107 | E1 | MF948920 | 99.71% |
| **OTU14** |  |  |  |  |  |  |
| 0 | 1446nt | PrPc079 | Pyr.3d | MF948937 | * | *Pseudomonas* sp. QND9 |
| **OTU15** |  |  |  |  |  |  |
| 0 | 1437nt | PrPc080 | Pyr.3d | MF948926 | 99.23% | *Pseudomonas pseudoalcaligenes* CH 1-2-2 |
| 1 | 1439nt | PrPx086 | Pyr.24d | MF948931 | 99.24% |
| 2 | 1441nt | PrPz072 | Pyr.30d | MF948933 | 99.24% |
| 3 | 1437nt | PrPf082 | Pyr.6d | MF948939 | 99.86% |
| 4 | 1446nt | PrPl084 | Pyr.12d | MF948929 | * | *Pseudomonas* sp. HNS021 |
| 5 | 1438nt | PrPy116 | E2 | MF948919 | 99.37% |
| 6 | 1440nt | PrPr088 | Pyr.18d | MF948930 | 99.10% | *Pseudomonas* sp. BP-1 |
| 7 | 1344nt | PrPy113 | E1 | MF948921 | 99.93% | *Pseudomonas* sp. MBEA06 |
| 8 | 1401nt | PrPy123 | E3 | MF948922 | 99.93% |
| **OTU16** |  |  |  |  |  |  |
| 0 | 1444nt | PrPb076 | Pyr.2d | MF948923 | * | *Pseudomonas putida* F2 |
| 1 | 1436nt | PrPf081 | Pyr.6d | MF948935 | 99.23% | *Pseudomonas* sp. GC04 |
| **OTU17** |  |  |  |  |  |  |
| 0 | 1441nt | PrP0029 | 2216E | MF948918 | 99.10% | *Pseudomonas monteilii*  WS14 |
| 1 | 1438nt | PrPc077 | Pyr.3d | MF948924 | 99.37% | *Pseudomonas putida* N-408 |
| 2 | 1442nt | PrPx087 | Pyr.24d | MF948932 | * | *Pseudomonas* sp. GC04 |
| **OTU18** |  |  |  |  |  |  |
| 0 | 1435nt | PrPb063 | Pyr.2d | MF948940 | 99.23% | *Acinetobacter* sp. HS-B1 |
| 1 | 1441nt | PrPb064 | Pyr.2d | MF948941 | * |
| **OTU19** |  |  |  |  |  |  |
| 0 | 1435nt | PrPc065 | Pyr.3d | MF948936 | 99.79% | *Acinetobacter venetianus* RMR 1 |
| 1 | 1441nt | PrPf069 | Pyr.6d | MF948938 | * |
| **OTU20** |  |  |  |  |  |  |
| 0 | 1440nt | PrP0027 | 2216E | MF948917 | * | *Acinetobacter* sp. HS-B1 |
| **OTU21** |  |  |  |  |  |  |
| 0 | 1440nt | PrPf067 | Pyr.6d | MF948943 | * | *Acinetobacter* sp. HS-B1 |
| **OTU22** |  |  |  |  |  |  |
| 0 | 1437nt | PrPc078 | Pyr.3d | MF948925 | * | *Pseudomonas* sp. YX10 |
| **OTU23** |  |  |  |  |  |  |
| 0 | 1436nt | PrP0028 | 2216E | MF948916 | * | *Pseudoalteromonas* sp. U1371-101227-XH108 |
| **OTU24** |  |  |  |  |  |  |
| 0 | 1436nt | PrPf083 | Pyr.6d | MF948934 | * | *Pseudomonas pseudoalcaligenes* RMR17 |
| **OTU25** |  |  |  |  |  |  |
| 0 | 1415nt | PrVy106 | E1 | MF948977 | 99.22% | *Vibrio hepatarius* VITPVA |
| 1 | 1384nt | PrVy111 | E1 | MF948983 | 99.57% | *Vibrio* sp. CR5 |
| 2 | 1417nt | PrVy120 | E3 | MF948976 | * |
| **OTU26** |  |  |  |  |  |  |
| 0 | 1393nt | PrRr055 | Pyr.18d | MF948954 | * | *Labrenzia* sp. SN-3-9-1 |
| 1 | 1374nt | PrRy136 | E1 | MF948952 | 99.49% | *Labrenzia* sp. ZJ2801 |
| **OTU27** |  |  |  |  |  |  |
| 0 | 1375nt | PrR0016 | 2216E | MF948946 | * | *Aliiroseovarius crassostreae* CV919-312 |
| **OTU28** |  |  |  |  |  |  |
| 0 | 1373nt | PrR0021 | 2216E | MF948948 | * | *Ruegeria* sp. LMD |
| **OTU29** |  |  |  |  |  |  |
| 0 | 1372nt | PrR0017 | 2216E | MF948947 | * | *Aliiroseovarius pelagivivens* GYSW-22 |
| **OTU30** |  |  |  |  |  |  |
| 0 | 1372nt | PrRf056 | Pyr.6d | MF948960 | * | *Yangia* sp. ND218 |
| **OTU31** |  |  |  |  |  |  |
| 0 | 1371nt | PrRz054 | Pyr.30d | MF948959 | * | *Citreicella* sp. KU27D1 |
| **OTU32** |  |  |  |  |  |  |
| 0 | 1370nt | PrR0018 | 2216E | MF948949 | * | *Roseovarius halocynthiae* MA1-10 |
| **OTU33** |  |  |  |  |  |  |
| 0 | 1368nt | PrRx057 | Pyr.24d | MF948956 | * | *Yangia* sp. QN187 |
| 1 | 1366nt | PrRx058 | Pyr.24d | MF948958 | 99.27% |
| **OTU34** |  |  |  |  |  |  |
| 0 | 1367nt | PrRr052 | Pyr.18d | MF948955 | * | *Roseobacter* sp. WHOI JT-01 |
| 1 | 1366nt | PrRx053 | Pyr.24d | MF948957 | 99.63% |
| **OTU35** |  |  |  |  |  |  |
| 0 | 1364nt | PrR0019 | 2216E | MF948950 | * | *Ruegeria pomeroyi* DSS-3 |
| **OTU36** |  |  |  |  |  |  |
| 0 | 1362nt | PrR0020 | 2216E | MF948951 | * | *Ruegeria* sp. B01Va |
| **OTU37** |  |  |  |  |  |  |
| 0 | 1327nt | PrRy144 | E2 | MF948953 | * | *Donghicola* sp. OG05 |

SUPPLEMENTARY TABLE S3 | OTUs defined by NGS sequences (Cutoff Value = 0.99).

| Samples | OTU_Count | OTU_Count | OTU_Count |
| --- | --- | --- | --- |
|  | Pseudomonadales | Vibrionales | Rhodobacterales |
| 0 d | 36 | 29 | 150 |
| 2 d | 563 | 1782 | 1217 |
| 3 d | 474 | 903 | 1358 |
| 6 d | 588 | 556 | 936 |
| 12 d | 50 | 257 | 266 |
| 18 d | 69 | 232 | 416 |
| 24 d | 45 | 144 | 788 |
| 30 d | 79 | 191 | 763 |
| Total | 2041 | 8863 | 5267 |

SUPPLEMENTARY TABLE S4 | Accession and description of 16S high-through sequencing data.

| Samples | Original_Samples_Name | Accession | Sample_Description |
| --- | --- | --- | --- |
| 0 d | 0 d | SRR6426925 | Estuarine sediment |
| 12 d | 12s1 | SRR6426921 | Domesticated cultures with pyrene |
|  | 12s2 | SRR6426920 |  |
|  | 12s3 | SRR6426923 |  |
| 18 d | 18s1 | SRR6426947 |  |
|  | 18s2 | SRR6426946 |  |
|  | 18s3 | SRR6426945 |  |
| 24 d | 24s1 | SRR6426963 |  |
|  | 24s2 | SRR6426964 |  |
|  | 24s3 | SRR6426961 |  |
| 2 d | 2s1 | SRR6426957 |  |
|  | 2s2 | SRR6426958 |  |
|  | 2s3 | SRR6426937 |  |
| 30 d | 30s1 | SRR6426933 |  |
|  | 30s2 | SRR6426932 |  |
|  | 30s3 | SRR6426935 |  |
| 3 d | 3s1 | SRR6426955 |  |
|  | 3s2 | SRR6426956 |  |
|  | 3s3 | SRR6426950 |  |
| 6 d | 6s1 | SRR6426953 |  |
|  | 6s2 | SRR6426954 |  |
|  | 6s3 | SRR6426918 |  |
